# Conditions for production of interdisciplinary teamwork outcomes in oncology teams: protocol for a realist evaluation

**DOI:** 10.1186/1748-5908-9-76

**Published:** 2014-06-17

**Authors:** Dominique Tremblay, Nassera Touati, Danièle Roberge, Jean-Louis Denis, Annie Turcotte, Benoît Samson

**Affiliations:** 1 Charles-Le Moyne Hospital Research Centre, Greenfield Park, QC J4K 0A8, Canada; 2 Faculty of Medicine and Health Sciences, Université de Sherbrooke-Campus Longueuil, Longueuil, QC J4K 0A8, Canada; 3 École Nationale d’Administration Publique, Montréal, QC G1K 9E5, Canada; 4 CSSS Champlain–Charles-Le Moyne, Longueuil, QC J4V 2H1, Canada

**Keywords:** Interdisciplinarity, Professional practices, Realist evaluation, Patient outcomes, Case study, Cancer

## Abstract

**Background:**

Interdisciplinary teamwork (ITW) is designed to promote the active participation of several disciplines in delivering comprehensive cancer care to patients. ITW provides mechanisms to support continuous communication among care providers, optimize professionals’ participation in clinical decision-making within and across disciplines, and foster care coordination along the cancer trajectory. However, ITW mechanisms are not activated optimally by all teams, resulting in a gap between desired outcomes of ITW and actual outcomes observed. The aim of the present study is to identify the conditions underlying outcome production by ITW in local oncology teams.

**Methods:**

This retrospective multiple case study will draw upon realist evaluation principles to explore associations among context, mechanisms and outcomes (CMO). The cases are nine interdisciplinary cancer teams that participated in a previous study evaluating ITW outcomes. Qualitative data sources will be used to construct a picture of CMO associations in each case. For data collection, reflexive focus groups will be held to capture patients’ and professionals’ perspectives on ITW, using the guiding question, ‘What works, for whom, and under what circumstances?’ Intra-case analysis will be used to trace associations between context, ITW mechanisms, and patient outcomes. Inter-case analysis will be used to compare the different cases’ CMO associations for a better understanding of the phenomenon under study.

**Discussion:**

This multiple case study will use realist evaluation principles to draw lessons about how certain contexts are more or less likely to produce particular outcomes. The results will make it possible to target more specifically the actions required to optimize structures and to activate the best mechanisms to meet the needs of cancer patients. This project could also contribute significantly to the development of improved research methods for conducting realist evaluations of complex healthcare interventions. To our knowledge, this study is the first to use CMO associations to improved empirical and theoretical understanding of interdisciplinary teamwork in oncology, and its results could foster more effective implementation in clinical practice.

## Background

Given the nature of their illness and its multimodal treatment, persons with cancer receive care from multiple professionals from different disciplines, working in a variety of settings, whose services may be provided either concurrently or at different points in time [1,2]. They are particularly susceptible to problems of fragmented or poorly coordinated care, communication failures, and breakdowns in care continuity, all of which compromise their timely access to needed care [3]. This results in unmet healthcare needs, with potentially harmful repercussions on patients’ health and well-being [4,5]. To address these deficiencies deplored by persons with cancer and their families, clinicians, and decision-makers, several countries such as Canada, the United Kingdom, the United States, and Australia have made interdisciplinary teamwork (ITW) a key element in their cancer care programs [6].

### Research opportunity

Quebec’s more than eight million residents enjoy a system of universal access to healthcare. The Quebec Cancer Program (PQLC – Programme québécois de lutte contre le cancer), publicly launched in 1998, was intended particularly to remedy deficiencies in service organization that had negative impacts both on the response to patients’ and families’ needs and on the quality and efficiency of care [7]. Since then, strategic plans have given priority to service reorganization, with a view to improving accessibility, continuity, person-centred care, and quality of care. This transformation has been supported by measures to promote better health services coordination and responsiveness. These include the creation of interdisciplinary teams in oncology outpatient clinics, the deployment of pivot nurses (also called nurse navigators in some parts of Canada), and the adoption of a person-centred approach [8]. The principal investigator of the proposed study and several members of the research team have conducted extensive research on health services transformation [9-16].

The PQLC, as other international cancer programs, supports the implementation of a strategy that would combine the efforts of all partners to produce the best possible response to the needs of persons with cancer. The PQLC’s 2007 to 2012 policy priorities recommended focusing on certain areas of intervention. ITW was presented as a solution to address the complexity of care and services required to meet the needs of persons with cancer [17].

Numerous efforts have been made and significant resources invested to encourage as many local oncology teams as possible to respond to these expectations. However, as we describe in greater detail below, the conclusions from evaluations of oncology care and services have clearly shown that ITW models vary from one team to another [3,8,18], as do the expected impacts on the care experience [9].

Generally speaking, studies on ITW (also called teamwork, collaborative practice, interprofessional practice) have concluded it produces benefits in terms of quality of patient care [19-24]. A systematic review of the literature showed that better interprofessional and interorganizational collaboration could be associated with positive outcomes, both clinically (*e.g*., reduced mortality, shorter hospital stays, fewer readmissions) and in terms of patients’ perceptions of their care experience (*e.g*., greater satisfaction, improved quality of life) [25]. However, in-depth critical analysis of the evidence provides a more nuanced assessment of the causal links between ITW and its effects on the care experience. The term ‘care experience’ refers to patients’ perceptions regarding key aspects of oncology care and services, such as access to services, the quality of the response to their needs, and the effectiveness of care when they interact with service providers [26].

There are several reasons justifying the need for a more thorough study of the conditions underlying the production of ITW outcomes as they relate to the care experience. First, the concept of interdisciplinary teamwork goes by several different names (*e.g*., multidisciplinarity, interdisciplinary teamwork, collaborative practice, interprofessional collaboration), which are used interchangeably but for which there are subtle differences of definition [27,28]. Because the subject being examined is not clearly defined and varies from one study to another, the result is a non-homogeneous body of knowledge from which it would seem rash to draw general conclusions on ITW outcomes for patients. Second, most of the instruments used in these studies to measure ITW focus on collaboration between physicians and nurses, whereas the outcomes may be different for teams with a more diverse professional composition [29], as is usually the case in oncology teams in Quebec. Third, some conclusions regarding outcomes related to ITW are also drawn from studies having to do with cancer therapy review committees (‘tumour boards’) [30,31], or else with interdisciplinary teams of specialist physicians (oncologists, pathologists, internists, surgeons) [32]. These teams focus on treatment options according to the model of consultation among physicians in different specialties rather than on comprehensive management of the continuum of care based on a whole-person care practice model [33]. Consequently, the outcomes measured in the participants in these studies have to do only with cancer treatments, rather than with the full experience of care including treatments, as envisioned by the PQLC. Finally, most of these studies provide very little information on the characteristics of the teams and their operational models, whereas ITW is known to be context-dependent [34]. One common conclusion emerges from the literature review, which is that, regardless of the type of interdisciplinary team considered, the mechanisms by which ITW produces specific outcomes in oncology, as they emerge in natural settings, are currently receiving very little research attention and are poorly understood [25].

The proposed study will build upon a quasi-experimental study recently conducted in nine Quebec hospitals with 1,379 patients (referred to as Study 1 in this proposal). The results of that study revealed significant differences in certain ITW outcomes for patients according to the level of interdisciplinarity (high vs. low) in local teams [9]. These differences had to do with the time elapsed before accessing a pivot nurse, the ability to contact the team when a need arose, the possibility of being seen when there is a deterioration in health status, the response to overall needs, assistance and involvement with care decisions, and feelings of anxiety and distress. Using multivariate regression analysis, Study 1 also revealed that individual factors (*e.g*. male gender, age 70 years and over, lower education level, positive perception of one’s health status) and organizational factors (*e.g.* receiving care from a team consisting of eight different professionals or fewer, receiving care in a rural-area clinic) were associated with a more positive patient care experience. These results confirm that certain teams in Quebec manage to produce some of the desired outcomes of ITW, while others are less successful. However, given its quasi-experimental design [10], Study 1 was not able to capture the factors and sequencing of processes that would explain the variations in outcomes. Given the current lack of knowledge, the results of Study 1, and the efforts being made to promote and support ITW in local oncology teams in Quebec, it is becoming imperative to better understand the mechanisms by which outcomes are produced, by whom, for what groups of patients, and in what contexts.

The aim of the present study is to map out the conditions for the production of ITW outcomes in local oncology clinics in Quebec. More specifically, the objectives are: a) to identify the most critical contextual factors and mechanisms associated with production of ITW patient outcomes; b) to determine the synergetic or antagonistic influences of contextual factors and mechanisms on the production of ITW patient outcomes; and c) to explore the occurrence of unanticipated ITW mechanisms and patient outcomes.

### ITW in oncology teams: intervention theory

Intervention theory refers to how the actors involved conceive of ITW in oncology teams, its functioning and its outcomes, as proposed by the PQLC. As part of the PQLC’s implementation, all local oncology teams in Quebec are expected to become involved in optimizing their ITW [11,12,17]. The aim is to ensure equity of access, without undue delays, to good-quality and safe care all along the cancer care continuum, from the first suspicion of illness to the period of survival or family bereavement, encompassing the entire period of active treatment and palliative care. ITW is a means of responding to complex and changing needs (of patients, families, and populations) by providing customized care centred on the whole person, at the right time, by the most appropriate professional, who is part of an oncology team. ITW relies on ‘synergy among practitioners (1 + 1 = 3)’ in a context of scarce resources, where the contributions of all professionals need to be optimized [13,17]. Additional file 1 contains more details for describing the intervention (ITW in oncology teams) in order to improve the completeness of reporting and ultimately the transferability of the study results.

### ITW in clinical teams: patient outcomes

In addition to the outcomes targeted by the PQLC, there are a number of other potential ITW outcomes identified in the literature. Despite the previously mentioned conceptual nuances characterizing the various terms applied to ITW, for the purposes of the following literature review we have adopted a pragmatic approach in which we consider them to be similar.

Overall, several studies have shown that ITW is associated with benefits for persons with chronic illnesses [14-16,35]. The results cover a wide range of observed outcomes, such as fewer clinical errors, complications, and hospitalizations, and shorter lengths of stay [36-39]. Various studies also report positive impacts of ITW on patients’ and families’ satisfaction with services [40,41], maintenance of functional capacity [40,42-44], compliance with treatment [44], greater accessibility to care, improved capacity for self-care, and healthy living habits, [43], health-promoting behaviours [43], and perceptions of better quality of care [45]. According to Schmitt and colleagues [43], ITW produces positive outcomes because it fosters a whole-person approach to the management of care. Other authors have associated positive outcomes with improved mechanisms for communication among professionals, who are increasingly working together toward a common goal [46]. However, there is no evidence that these benefits observed in other clienteles are transferable to patients with cancer. We also did not find any negative patient outcomes associated with ITW.

More specifically in oncology, a study in the United Kingdom explored the care experience of adolescents with cancer who were treated by an interdisciplinary team [47]. One conclusion of that qualitative study was that ITW in dedicated and specialized teams is a key element in creating a care environment that is responsive to the complex needs of this clientele. More recently, a study in a hospital in Spain showed that a higher level of ITW in the oncology care units was significantly associated with better pain management, higher levels of satisfaction among patients, and reduced uncertainty related to their illness [35]. That same study concluded that the intensity of collaboration had no impact on length of hospital stay. Our own work in Study 1 showed that ITW outcomes varied from one team to another. In some teams, the results confirmed our initial hypothesis of positive covariance between ITW level and the scope of effects for patients, *i.e*., that higher ITW levels would be accompanied by more positive patient perceptions of its effects on their experience. However, we found that, for certain aspects of the care experience, teams with lower ITW levels obtained results that were just as positive as those with higher ITW levels. Our results also showed that teams with a high level of ITW achieved outcomes that were less substantial than expected. While it is not clear why or how, it appears there may be one or more intermediate factors acting as moderator or mediator in the relationship between ITW and patient outcomes.

We should also point out that our results align with the conclusions of other authors regarding the need for further studies to better understand the links between ITW and its effects on quality of care. A recent review of the literature on ITW in oncology [33] reached the same conclusion as Lemieux-Charles and colleagues in 2006 [23], which was that studies establishing links between quality of team functioning and clinical effectiveness are few and far between, and offer inconsistent results. Some authors have criticized scientific journals’ tendency to publish only studies showing positive results of ITW [34], which constitutes a publication bias; they note the real absence of studies that observed either no effects or negative effects of ITW. According to these authors, this bias has meant that ITW is becoming a standard that is less and less subject to critical analysis. They also point out that many literature reviews on ITW in care teams have concluded that outcomes are positive without having evaluated the quality of the studies. Assessing the quality of the studies might reveal that the outcomes are less robust than is generally thought. There is also evidence that intra- and interprofessional collaborative practice is a best practice associated with credible patient outcomes [24]. Thus, given its nature and its effects, ITW can be approached as a complex intervention. According to the Medical Research Council, a complex intervention is characterized by several interdependent components, involves the practices of several actors in the healthcare system, and produces a variety of effects whose nature and scope can vary depending on the context [48]. Several authors agree that exploring these interventions more deeply presents very particular conceptual and methodological challenges [49-51].

### ITW in oncology teams: the challenges of evaluating complex interventions

The ‘realistic evaluation’ approach developed by Pawson and Tilley [52] is recognized as being useful for studying complex interventions when the aim is to go beyond determining whether an intervention is effective or not, and instead to explain how and why it is effective, under what conditions, and for which groups of patients. To be a ‘realistic evaluation’, the approach must be able to capture the contextualized action mechanisms at work in the relationship between an intervention and its effects, and to identify how these mechanisms are activated (or not). A mechanism refers to all ‘underlying entities, processes, or structures which operate in particular contexts to generate outcomes of interest’ [53]. Realistic evaluation is based on the theory: intervention context (C) + mechanism (M) = outcome (O), analyzed using a configurational approach. CMO configurations serve as a structure to identify what works (or not), how, by whom, with whom, for whom, and in what context. This theory is appropriate to guide our study because it links the conditions of production (C + M) and the outcomes (O) of an intervention according to a structured evaluation approach to what is actually happening in natural environments. It offers the possibility of deepening our understanding of ITW outcomes in oncology by identifying the synergetic or antagonistic effects in these associations among various contextual factors and action mechanisms that either foster or hinder the production of outcomes (Objectives 1 and 2 of the present study). The realistic evaluation approach is a pragmatic alternative to the experimental paradigm, given the impossibility of controlling complex interventions in natural environments, such as ITW [54]. Realistic evaluation thus offers a systematic investigative structure with two key advantages: it can take into account a multiplicity of variables and their relationships implied in the production of an intervention’s outcomes; and it can be used, at the same time, to minimize this complexity by modelling the relationships based on configurations of the three main dimensions: context–mechanisms–outcomes; both of these advantages facilitate the investigation and support potential action by users of the study’s results. Thus, contextual factors are considered not as variables to be controlled, as would be the case in experimental studies, but rather as intrinsic components of the phenomenon under study.

In addition to these theoretical foundations, realistic evaluation provides five key principles to consider in the research process: a) stakeholder participation in a study is required to understand – realistically – CMO configurations; b) mechanisms are theories developed based on propositions that ‘if things work this way in a given context, these will be the results’; c) CMO configurations may be established at the start, but they will be refined throughout the course of the study; d) causal links are generated as the study unfolds by determining how the variables are associated, rather than simply by measuring the correlation between a variable and a given outcome (as was done in Study 1); and e) the expected outcomes of an intervention are never guaranteed because of the central role played by the context [54,55]. These realistic evaluation principles suggest that the first thing to do is to target the mechanisms that are likely to be active in the phenomenon before starting the study. In the following sections, realistic evaluation concepts are used to identify the contextual factors and mechanisms that are potentially involved in the production of ITW outcomes.

### ITW in oncology teams: contextual factors

Those researchers who have a critical approach to ITW agree on the importance of context in explaining its variations. Context refers to the immediate and more distant environments whose structural characteristics shape care delivery [56]. As such, it is important to analyze context dynamically. Several contextual factors can contribute to the effectiveness of teams’ functioning [23,57,58]. These factors fall into three groups defined according to decision-making levels in the health system. The first group is at the practice (micro) level and encompasses the characteristics of professionals (*e.g*., profession, training, experience, leadership), patients’ clinical and sociodemographic characteristics (*e.g*., age, type of cancer, type of treatment, general health status, comorbidities), and team characteristics (*e.g*., composition, size, nature of mandate, work climate) [59-61]. The second group is at the organizational (meso) level and includes institutional support, access to resources, leadership, etc. [15]. The third group is at the systemic (macro) level and comprises policies, national programs, legislative frameworks governing professional practice, etc. [62]. Each of these factors can exert variable influence in different CMO configurations, influences that will be more clearly delineated by the results of this present study.

### ITW in oncology teams: mechanisms associated with outcomes production

Based on the ITW attributes described in the intervention theory coming out of the PQLC documents, it is possible to identify certain mechanisms that are potentially associated with the production of ITW outcomes in oncology. The first attribute – the bringing together of several practitioners – presents the challenge of sharing in the group the expertise, knowledge, and experience of professionals from different disciplines, who each have their own specific skills [34]. For a group of heterogeneous practitioners to rally around a common goal and collaborate, an effective communication mechanism must be in place and active [25,57,63-66]. The second attribute – working together – requires a mechanism for care coordination to ensure continuity of care [25,66]. When coordination is active, team members function interdependently, with each person’s contribution being important to optimize the management of information, clinical processes, and resources [67]. The third attribute – a global, shared, and unified understanding of the person – introduces the mechanism of patient-centred care [68]. Thus, instead of fragmented services centred around issues of professionalization, patients are presented with a person-centred care model in which an interdisciplinary care plan can be developed with objectives shared by the different practitioners on the team [69]. The fourth attribute –a concerted intervention that involves the sharing of complementary tasks – can refer both to a collaboration mechanism within the team and to integration between organizations [28,70]. Finally, a fifth attribute appears to be essential for optimizing outcomes – evidence-based practice supported by a scientific knowledge utilization mechanism. Given the multidimensional nature of each of these mechanisms, it is worthwhile to characterize them here more clearly and to identify their potential effects based on the literature (see Additional file 2 for a detailed description and references).

### ITW in oncology teams: interpretive framework

**Figure 1:**
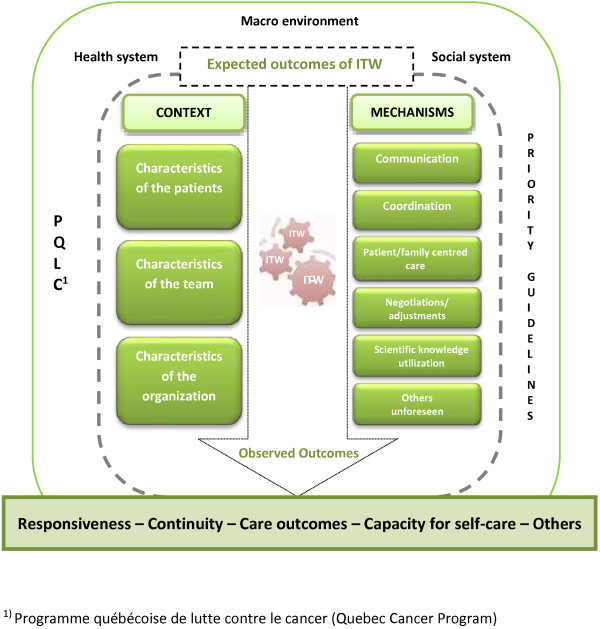
Interpretive framework for evaluating the conditions for the production of ITW outcomes in oncology. ^1)^ Programme québécoise de lutte contre le cancer (Quebec Cancer Program).

The intervention (ITW) coming out of the PQLC documents, the literature on ITW (definition, facilitating factors, constraining factors, outcomes), and the dimensions of realistic evaluation brings us to develop an interpretive framework (Figure 1).

The framework thus incorporates three broad components surrounding ITW: a) the context, including characteristics of the environment, characteristics of the oncology clientele that could have an influence on the organization of the continuum of care and services, and characteristics of the organization and the team; b) mechanisms put forward to achieve ITW outcomes (communication, coordination, collaboration, person-centred care, negotiations and mutual adjustments, scientific knowledge utilization, and others that may be unforeseen); and c) the expected outcomes of ITW based on the intervention theory set out in the PQLC and the policy priorities as promised by the Quebec’s Department of Cancer Services. Using the data from Study 1, we will analyze the contextual factors and the mechanisms (C + M) associated with certain outcomes of interdisciplinarity that are comprised of several dimensions. We will analyze: responsiveness [71], which can be broken down into four subdimensions (promptness of access to care in response to patients’ needs, and the quality of communication, of the care environment, and of the person-centred response); continuity of care [72]; care outcomes [73] (management of symptoms and of health), as well as two subscales of the questionnaire on the capacity for self-care [74] (well-being and navigation of the healthcare system).

In light of these points, the key theories to be explored initially are:

1. Initial theory: that there is a positive relationship between ITW and certain aspects of the care experience as perceived by patients.

2. Alternative theory A: that there is a synergy (1 + 1 = 3) between certain contextual factors and certain mechanisms of team functioning that have a mediating influence on the relationship between ITW in oncology and patients’ perception of certain aspects of their care experience.

3. Alternative theory B: that there is an antagonistic association (1 + 1 = 1) between certain contextual factors and certain mechanisms of team functioning that have a moderating influence on the relationship between ITW in oncology and patients’ perception of certain aspects of their care experience.

## Methods

### Design

The study will use a retrospective multiple case study (n = 9) design [75]. Case study is an appropriate approach for characterizing clinical and organizational processes in their contexts [75-77]. This is, in fact, the study design most often used by researchers investigating CMO configurations in the healthcare field [78-81]. Each case consists of an interdisciplinary team in oncology working in the ambulatory clinic of a hospital. We will perform intra-case analyses to identify the CMO configurations, and then a cross-cutting inter-case analysis [82] to compare the cases. The study is retrospective, in that we will proceed in reverse to reconstruct the C + M associations that explain the outcomes measured in Study 1 (responsiveness, continuity, care outcomes, capacity for self-care) [83].

### Case selection

The nine sites that participated in Study 1 will be invited to participate in the present study. For a multiple case study, the aim is not to have a sample that is statistically representative of a population, but rather to have the most informative cases possible, in which the features observed are likely to exist in other cases of populations with similar characteristics [76,82,84]. The diversity of the cases studied will allow us to observe recursive or singular CMO configuration models and to draw conclusions that could potentially be transferable to the different teams across Quebec.

### Participants

The data, which will be qualitative, will be collected primarily by means of focus groups [85]. For each site, two different participant groups will be formed: G1 will be made up of the professionals in the oncology care team and the managers directly involved in the team’s deployment of ITW; G2 will consist of patients who have received services from that team. The number of participants per group will be around 6 to 10, perhaps going as high as 15 in certain cases, depending on the size of the local oncology team [86]. The criterion for inclusion in G1 is membership on the team for at least the past three years, to be able to provide an evolutionary perspective of the context and mechanisms put forward to support ITW since the start of Study 1. The criteria for inclusion in G2 (patients) are: a) to be comfortable with participating in a group discussion, and b) to have been in active treatment during the past year, such that they have had enough ‘exposure’ to the interdisciplinary team to be able to draw some conclusions about their care experience. They will be purposefully selected, with the help of a member of the local team, to ensure a diversity of sociodemographic characteristics (sex, age, education) and of clinical characteristics (type of treatment, type of cancer, phase of illness) [87]. The focus group approach was a strategic choice. It allows for spontaneous but focused discussion to better understand the conditions for the production of ITW outcomes from the perspectives of the persons most directly concerned. Focus groups provoke a ‘good conversation’ on the phenomenon being studied and make the best use of the limited time available to the group for in-depth consideration of the subject [88]. Particular attention will be paid to five criteria for assessing the success of a focus group: a) the intended topics are covered, and in sufficient depth; b) the statements elicited provide rich, diverse and nuanced testimony; c) participation is active, spontaneous and balanced; d) there is interaction and mutual influence, but no excessive dominance by any participants; and e) the group process contributes to reflection that allows participants to embark in a change process [85]. In this way, we will be able to cover all of the potential explanatory factors for the production of ITW outcomes by starting from the outcomes and working backwards to investigate the conditions (context and mechanisms) that determine them [83] (Objectives 1 and 2), while the spontaneity and dynamic nature of the group will enrich the explanations and help to identify unanticipated mechanisms and outcomes (Objective 3).

The focus group data will be triangulated with complementary data gleaned from the relevant documentation (meeting minutes, medical records charting tools, inter-professional and inter-organizational referral tools) and from informal discussions [82].

### Data collection

Data will be systematically collected in the focus groups with professionals and patients by means of pre-defined questions presented in the focus group meeting plan (see Additional file 3). The groups will be facilitated by the co-investigators, who have extensive experience in oncology and in leading oncology team meetings. The professionals will be invited to reflect on the conditions that might explain the ITW outcomes in their setting. These outcomes, measured in Study 1, are presented in the local reports distributed to the teams in 2012. Those reports summarize the context of Study 1 and its methodology, and present descriptive statistics from the responses to the questionnaire on accessibility, responsiveness, continuity, capacity for self-care, and characteristics of respondents. The report presenting both local and global results (aggregated for the nine sites) will be used as an intermediary object [89] in a reflexive process (learning about and in practice) [90] and as a means of encouraging discussion. For items that characterize the greatest variation in the care experience of patients receiving care from the local team, the professionals will take a position on what they think about the results, as well as on the gaps between local and global results and how they might be explained: what mechanisms facilitate the production of ITW outcomes; what mechanisms contribute to maintaining practices that are less interdisciplinary; and by whom, with whom, and for whom outcomes are produced, and in what specific contexts.

With regard to the patient focus groups, the local report is considered too abstract to provoke any discussion on the care experience. As such, the intermediary object chosen for that purpose will be a vignette covering the active treatment period. Used in research, a vignette is a brief scenario presented either in writing or through pictograms, which serves as a proxy for a real situation, and to which participants are invited to respond [91]. The vignette has several advantages: it helps depersonalize the discussion while keeping the focus on the phenomenon under study, and it is a standardized, flexible, and inexpensive tool. As we did in an earlier study to assess the quality of care in oncology [92], we will reconstruct the cancer care continuum of a patient with colon cancer (post-surgery diagnosis, first phase of chemotherapy, progression of the illness, and decision to pursue more aggressive treatment), inserting certain items that showed the greatest variation in ITW outcomes when measured in Study 1. For example, the vignette will address items related to responsiveness by illustrating such things as participation in decisions about care, help in weighing the pros and cons of treatment, family involvement, and support in managing anxiety when dealing with illness.

The questions used to structure the discussion are inspired by realistic evaluation and are aimed not only at identifying ITW outcomes (the ‘what’), but also at explaining how and why those outcomes are produced. At each focus group meeting, we will explain the process we intend to follow, then present a summary of the results of Study 1, followed by a discussion as presented in the focus group outline provided in the Additional file 3[93]. The discussions will be digitally recorded and then transcribed in their entirety for analysis. All the material will be entered into a database using QDA Miner software [94].

### Data analysis

The data will be analyzed using an iterative content analysis process with systematic coding [95,96]. The analysis will be structured based on the 10 steps developed by Ely [97]. First, we will develop a semi-structured coding grid based on the concepts of our interpretive framework (context, mechanisms, outcomes) leaving open the possibility of adding emerging concepts to the CMO configurations over the course of the analysis [98]. The data for each case will be analyzed separately. Then we will perform an inter-case analysis to identify similar and different CMO configurations in the different cases. According to Campbell [99], certain case characteristics may only become evident through inter-case comparisons. Finally, we will use a process of iteration between the intra-case and inter-cases analyses to ensure that we are able to satisfy the objectives of the study and to produce enough evidence to facilitate the transferability of our results to other organizations whose characteristics are similar to those in our study cases. To ensure the quality of the analysis, the members of the research team will discuss the coding process and results among themselves and also with collaborators and key stakeholders (members of professional teams, managers, decision-makers) in validation meetings. If we are able to identify patients who would be at ease and willing to participate in such validation discussions, they will be included.

The study has been approved by the Research Ethics Board of the Charles-LeMoyne Hospital Research Centre (ref. number MP-HCLM-13-034).

### Study validity

In this study, we will pay special attention to ensuring both internal and external validity. Internal validity refers to the degree of similarity and plausibility that exists between the knowledge construction process, the complexity of a phenomenon in its natural setting, and the results of a study [95]. We have employed several means to ensure the internal validity of our study: using cases whose characteristics we know well from having worked with them in Study 1 [75,83]; incorporating an extensive review of the ITW literature; building on the theoretical foundations and methodologies of realistic evaluation [100]; and triangulating several data sources [101], *i.e*., the perspectives of professionals and patients, as well as documentary analysis. We will triangulate data sources using two strategies that highlight different aspects of the conditions for production of ITW outcomes: a) the comparison of similarities and differences in the perspectives of the various actors with regard to the context (C) and mechanisms (M) of interdisciplinary care and the C + M links in the proposed study; and b) the validation of data from the interviews as compared with the data from documentary sources [87]. In our proposed study, the solidity of the links established between context, mechanisms and outcomes is reinforced by the study of multiple cases [82] and by the complementarity of the quantitative and qualitative research approaches of Study 1 and the present study [102,103]. Moreover, Rohlfing (2008) asserts that, by taking outcomes as the starting point and working backward to investigate the conditions that determine them, we are more likely to capture the whole range of causes that might have influenced the outcomes [83]. With regard to external validity, it has to do with the degree to which a study’s results can be generalized to other populations, settings, and time periods having similar characteristics [104]. However, case study, in the context of realistic evaluation, does not aim for quantitative or statistical generalizability, but rather to provide plausible explanations obtained from a sample in which, ‘if things work this way, these will be the results.’ These explanations can be generalized to other contexts where the organizational and environmental conditions are similar [95].

## Discussion

To the best of our knowledge, this study is the first to use CMO associations to improved empirical and theoretical understanding of interdisciplinary teamwork in oncology, and its results could foster more effective implementation in clinical practice. This study will have significant impacts because the results will foster innovation by helping to fill the gaps in knowledge about the strongly contextualized mechanisms responsible for differences in ITW outcomes in oncology. It will be possible to identify which are the most critical mechanisms to activate, to determine whether certain mechanisms are more important than others, and to specify in which contexts these mechanisms are most active. With a better understanding of these factors, it will be possible to target interventions to act on the determining conditions in ways that optimize positive outcomes. Settings with less favourable conditions can be better supported in their efforts to optimize ITW. Given the study design, the results will be useful for different types of actors at different levels of the healthcare system, *i.e*., clinicians, managers, and decision-makers who determine strategic actions in the PQLC’s policy priorities. These new data will be especially important in a context where every professional’s contribution will need to be optimized, to contend with the rising incidence of cancer as the population ages and the limited resources of the healthcare system.

## Abbreviations

CMO: Context–mechanisms–outcomes; ITW: Interdisciplinary teamwork; PQLC: Programme québécois de lutte contre le cancer/Quebec cancer program.

## Competing interests

The authors declare that they have no competing interests.

## Authors’ contributions

DT led the coordination and the conceptualization of the study. DT and NT wrote the first draft, DR critically reviewed it and provided comments to improve the manuscript. All authors have read and commented on the final manuscript.

## Supplementary Material

Additional file 1 Description of the intervention: interdisciplinary teamwork (ITW) in oncology teams. Click here for file

Additional file 2 Description of the mechanisms involved in the production of interdisciplinary teamwork (ITW) outcomes. Click here for file

Additional file 3 Description of focus group meeting plan (professionals and patients). Click here for file
