# Supplementary material for: Conditions for production of interdisciplinary teamwork outcomes in oncology teams: protocol for a realist evaluation
Source: Implement Sci. 2014 Jun 17;9:76. doi: 10.1186/1748-5908-9-76 (PMC4074333; doi:10.1186/1748-5908-9-76)
Supplement: Additional file 1 — Description of the intervention: interdisciplinary teamwork (ITW) in oncology teams. [file 1748-5908-9-76-S1.doc]

**Additional file 1 – Description of the intervention: interdisciplinary teamwork (ITW) in oncology teams**

The overarching purpose of this additional file is to describe the intervention in sufficient detail to allow better understanding for international readers. Even if the study focuses on the province of Quebec, the intervention components are similar to other cancer programs internationally.

Interdisciplinary teamwork is a key element of the Quebec cancer program (PQLC – Programme québécois de lutte contre le cancer) launched in 1998 . The continuum of care is ensured through a hierarchical service organization consisting of three types of teams with mandates at different levels: local, regional, and supraregional. The present study focuses on local cancer teams. The Ministry of Health guidance on interdisciplinary teams in oncology refers to Hébert, who defines interdisciplinarity as “… bringing together several practitioners with specific training, competence, and experience, who work together to produce a global, shared, and unified understanding of the person, with a view to implementing a concerted intervention that involves the sharing of complementary tasks” . The authors of that guidance favoured this definition because it represents the type of team whose core focus is the person receiving care and whose goal is to develop a concerted care plan. This is the definition we have retained as the basis for the work of the present study.

The cancer action plan identifies certain aspects of context, operational model, and intended patient outcomes . The PQLC specifies that local teams should minimally consist of the following professionals: a dedicated oncology nurse, a pivot nurse in oncology (also referred to as nurse navigator or oncology coordinator in the literature), a hematologist-oncologist or medical oncologist, a nutritionist, a pharmacist, and either a psychologist or a social worker. These professionals should attend team meetings regularly. The operational model is based on a philosophy of shared care, a coordination mechanism with several key components: data collection by the pivot nurse, interdisciplinary meetings to discuss complex cases, team development of an interdisciplinary intervention plan, and a mechanism for collaboration among all partners providing oncology care and services. The rules of functioning should be spelled out, and roles should be clear and supported by effective communication processes. The targeted positive patient outcomes would be, for example: improved quality of care, greater accessibility, better assessment and management of symptoms, and more effective continuity of care. Other positive outcomes for patients include shorter waiting times for services, respect for their values and preferences of the patient, greater inclusion in decision-making, lower levels of distress, and greater recognition and use of the person’s competences, strengths, and resources.

The interdisciplinary teams described above are different from cancer therapy review committees, or tumour boards. The former are characterized by a holistic person-centred approach to care in which each professional makes a contribution, using specific coordination, communication, and coordination mechanisms to provide patients with personalized, cohesive care. The latter are essentially forums for discussion and consultation among medical specialists (e.g. surgeons, oncologists, pathologists, radio-oncologists) around tumour data, cancer staging, and medical treatment regimens . The Ministry of Health guidance specifies that, if the appropriate mechanisms are not activated, undesirable outcomes could occur that run counter to ITW objectives: interpersonal conflicts, power struggles, wasted time, frustration, defamation, ineffectiveness—all situations that can erode quality of care.

References
