# Supplementary material for: Conditions for production of interdisciplinary teamwork outcomes in oncology teams: protocol for a realist evaluation
Source: Implement Sci. 2014 Jun 17;9:76. doi: 10.1186/1748-5908-9-76 (PMC4074333; doi:10.1186/1748-5908-9-76)
Supplement: Additional file 2 — Description of the mechanisms involved in the production of interdisciplinary teamwork (ITW) outcomes. [file 1748-5908-9-76-S2.doc]

**Additional file 2 – Description of the mechanisms involved in the production of interdisciplinary teamwork (ITW) outcomes**

The following text presents a detailed description of potential mechanisms put forward to achieve ITW outcomes: communication, coordination, collaboration, person-centred care, negotiations and mutual adjustments, and scientific knowledge utilization. Empirical studies can be used to identify certain mechanisms that may be involved in the production of ITW outcomes in patients. Even if the mechanisms described below make sense in terms of the literature and in an intervention logic model, we do not know whether certain mechanisms are more critical than others, or whether they are all necessary or equally important for achieving the intended goals of ITW. Based on our discussions with persons involved in oncology care, the mechanisms presented above could help to improve the overall cancer care experience. However, there are still challenges involved in determining which strategies should be used to activate these mechanisms in teams, and our study aims to shed light on this matter.

Communication mechanism: A systematic literature review by Thorne (2005) showed the deleterious effects of poor communication between professionals working with persons with cancer . These effects included patients’ more frequent recourse to complementary and/or unnecessary therapies, poor pain management, higher levels of emotional distress, and lower levels of satisfaction.

In fields other than oncology, authors have demonstrated that lack of communication due to interpersonal conflict between two or more members of the care team could be associated with serious medical errors . Along the same lines, the Joint Commission in the United States considers poor communication to be a sentinel event because it is an underlying cause of serious accidents that could even lead to death . The mechanism of communication also introduces the notion of informational continuity, defined as the availability and use of information on events in previous episodes of care (other visits, test results, recommendations, or information services) . Studies that have examined the effects of informational continuity on the care experience have shown that physicians’ and nurses’ knowledge about the patient’s medical history appears to be linked with more effective and more personalized care .

Care coordination mechanism: Because interventions occur all along the cancer care continuum, some concurrently and others at different times and in different settings, professionals need mechanisms both for internal coordination and for coordination with others outside the care team, in order to achieve the care outcomes targeted for patients. Coordination refers to all the arrangements that enable the different parts of the whole to be logically organized. Alter and Hage describe three types of coordination: 1) sequential, involving successive meetings of professionals; 2) reciprocal, in which each professional must keep track of what the others are doing; and 3) collective, in which team members are jointly responsible for managing care . Coordination is an essential element in teams characterized by significant levels of specialization and differentiation among members , who practice in contexts where there is considerable uncertainty regarding patients’ responses to planned interventions. To minimize this uncertainty, formal coordination mechanisms must be activated. These could include, for example, data collection by the pivot nurse; interdisciplinary meetings were complex cases are discussed; development of an interdisciplinary intervention plan; collaboration with all care partners, from primary care to ultraspecialized care; and the use of evidence-based standardized care protocols .

Added to these formal coordination activities are informal activities such as ‘corridor’ discussions, the influence of opinion leaders , and feedback processes (discussion of care outcomes, adjustments to care plans) . Several studies have demonstrated the relationship between coordination and certain patient outcomes such as reduced length of stay, greater satisfaction, improved quality of care , lower rates of unexpected death , decreased morbidity , and reduced post-operative pain. Our previous studies have also shown that optimal deployment of the pivot nurse role, particularly for care coordination, was associated with greater responsiveness of services in oncology, especially when the first contact was established within the first two weeks after the cancer diagnosis .

Person-centred care mechanism (PCC): The concept of person-centred care, which emerged in the 1950s, has over time become a best practice that produces more positive patient outcomes . PCC is an approach that emphasizes the defense of patients’ rights, patient empowerment, and respect for patients’ autonomy, opinions, self-determination, and involvement in decision-making. PCC is considered to be a prerequisite for integrated care provision in the context of ITW practice in healthcare networks . A recent literature review linked the communication mechanism with that of PCC for persons with cancer. Activating PCC had positive effects for patients, such as greater capacity for self-care and improved ability to make decisions, manage their emotions, and establish positive relationships with professionals .

PCC underlies another process that is known to produce better care outcomes, which is shared decision-making between professionals and patients, in which decisional autonomy and capacity to choose form the basis of the therapeutic relationship. Shared decision-making has the potential to: 1) reduce over-utilization of treatments whose positive effects have not been clearly demonstrated in all cases (i.g. prostate cancer treatment); 2) increase the use of interventions whose benefits have been clearly demonstrated for everyone (i.g. smoking cessation); 3) decrease unjustified variations in care practices; and 4) recognize patients’ right to be involved in decisions about their own health . Our studies have also shown PCC to be a dimension of responsiveness in oncology services, with responsiveness being defined as the capacity to respond to patients’ needs .

Collaboration mechanism: Collaboration is a mechanism by which collective action can be structured to resolve issues of service fragmentation and duplication while also better controlling costs . Several processes are involved in establishing collaboration, most notably the redefinition of the boundaries delineating roles and tasks (of individuals or organizations) . There needs to be opportunities for negotiation and mutual adjustments among members of the care team, even outside of clinical team meetings. For example, Cott studied negotiation processes in describing the nature of hierarchies and ITW in rehabilitation teams . Svensson explored the nature of physician–nurse negotiations in decision-making around patient care and concluded that these negotiations involved equal-to-equal dynamics at the micro level that made it possible to organize well-functioning teams . A comparative study also demonstrated that physicians who accepted around 90% of recommendations made by pharmacists were significantly more likely to achieve targeted patient outcomes than were those who rejected the recommendations . Collaboration among practitioners working in different settings requires access to information technologies (electronic health records, results transmission), standardized treatment and referral protocols, and the use of practice guidelines that have been agreed to by all the professionals, in order to harmonize practices affecting the patient all along the cancer care continuum.

Scientific knowledge utilization mechanism: Having access to scientific evidence and being able to adapt it to the particular circumstances of a person’s situation are linked to the development of care protocols and practice guidelines, as well as to the development of outcomes indicators and to the reflexive approach to evaluating quality of care. This mechanism implies that team members will evaluate their practice, analyze their performance, and share their positive experiences of improving quality of care . A recent study by Rycroft-Malone showed that engaging in explicit discussions about scientific evidence led to more practice improvements than did just using the evidence implicitly in care protocols . Moreover, Greenhalgh and colleagues demonstrated that this mechanism could be constrained or facilitated depending on the quality of the evidence, the team’s ability to access it at the right time, and the presence of activities for feedback on the impacts of updating best practices . We should add that the exact ways in which context influences access to, and use of, evidence are still not very well understood .

It is thus possible to identify up front certain mechanisms involved in the production of ITW outcomes, as suggested by one of the principles of realistic evaluation. However, the research data on these mechanisms come from studies involving clienteles other than persons with cancer, and we do not know how transferable these data might be. It is also possible that an outcome could be caused by one or more mechanisms, activated concurrently or at different times, which is rarely document in the literature . In some cases, one mechanism may need to be activated first in order to activate another and produce the intended outcomes. For example, in order to provide personalized care that is responsive to the patients’ overall needs, evidence-based practice mechanisms need to be embedded in person-centred care mechanisms based on the person’s values and preferences. There is very little evidence on how these mechanisms are activated and on their combined effects in different contexts. It can also be difficult to differentiate among mechanisms of contextual factors .

**References**
