# Supplementary material for: Conditions for production of interdisciplinary teamwork outcomes in oncology teams: protocol for a realist evaluation
Source: Implement Sci. 2014 Jun 17;9:76. doi: 10.1186/1748-5908-9-76 (PMC4074333; doi:10.1186/1748-5908-9-76)
Supplement: Additional file 3 — Description of focus group meeting plan (professionals and patients). [file 1748-5908-9-76-S3.docx]

## Additional file 3 – Description of focus group meeting plan (professionals and patients)

**Focus groups with professionals**

**A) Objective**: Based on the results of Study 1 presented in the local report, the professionals will be invited to identify the contextual factors and mechanisms associated with ITW outcomes. They will also be invited to identify other outcomes not measured in Study 1.

**B) Procedure (120 minutes)**

**Step 1: Introduction and creation of a climate of trust**

• Review of the objectives of Study 1

• Description of the broad sections of the questionnaire for patients

• Description of the local outcomes compared with the aggregated outcomes of all the sites

**Step 2: Discussion led by the facilitator**

Step 2 involves a structured discussion among the professionals to reflect on the nature and scope of the outcomes. The facilitator will ask the questions so that the criteria for good focus group conduct will be respected by creating a climate of trust and respect.

Specific questions on context, mechanisms, and outcomes, and how they are related:

- What do you think of the results presented in the report?
- In what ways do they resemble what you think or observe in your practice?
- How are they different or surprising?
- Why did this outcome (name each of the outcomes that present a difference, one after the other) produce a more positive (or negative) care experience for the greatest proportion of patients?
- By whom are these outcomes mainly produced?
- What operational mechanisms facilitate the production of these outcomes? By mechanisms, we mean….)
- If the responses do not emerge spontaneously, probe the mechanisms presented in the interpretive framework
- Which mechanisms get in the way of achieving outcomes in the way you would like?
- What does your team have that facilitates the achievement of (name an outcome)?
- What does your team have that makes achieving (name an outcome) more difficult?
- What does your organization, your regional network, have that facilitates the achievement of (name an outcome)? What does your organization, your regional network, have that makes achieving (name an outcome) more difficult?
- Do you see other ITW outcomes that are not presented in the report?
- What CMO association(s) is (are) the most critical to enable your team to more effectively improve the response to patients’ needs?
- What action can you take – individually or collectively – to optimize this (these) CMO(s)?


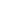
**C) End of the focus group discussion**

- Thanks to everyone for their participation
- Reminder about confidentiality
- Designation of one person to represent the group in follow-up with the research team

**Focus groups with patients**

**A) Objective**: From a vignette illustrating the local ITW outcomes obtained in Study 1, patients will be invited to identify what is happening to a fictional patient with colon cancer, whom we have called Mr. Gauthier. A scenario will be presented that illustrates three points in his experience of the cancer care continuum: 1) receiving news of the diagnosis; 2) visits to the oncology clinic during active treatment; and 3) participation in decision-making regarding a more advanced line of treatment. This strategy is known to be useful for the systematic collection of information on a phenomenon while respecting the private lives of participants in a group activity.

**B) Procedure (120 minutes)**

**Step 1: Introduction and creation of a climate of trust**

• Review of the objectives of Study 1

• Description of the broad sections of the questionnaire for patients

• Description of the local outcomes compared with the aggregated outcomes of all the sites

**Step 2: Discussion led by the facilitator**

Step 2 will allow patients to talk about the aspects of the fictional patient’s care experience in relation to what they themselves observed of the care generally provided in their setting. The facilitator will support the process by creating a climate based on trust and respect.

Specific questions for each stage of the vignette: For each of the four stages described in the vignette:

- What do you see in Mr. Gauthier’s (name the stage of the care experience)? – In what way is it similar to what happens here at (name the site)?
- Is there anything that seems different or surprising to you, compared to what happens here at (name the site)?
- Is there anything that could have been done differently so that things could go as well as possible for Mr. Gauthier?
- By whom? With whom?
- What does Mr. Gauthier do to see whether the professionals are working together to respond to his needs?
- Probe whether the professionals are up-to-date on his recent status, whether they talk with each other, whether they send results to the family physician, to the CLSC
- What should Mr. Gauthier do in order to talk with a professional about the emergence of a problem with pain and redness in his mouth?
- Probe whether the patients know where to call, link with pivot nurse, accessibility in cases of need, possibility of being seen when the needed arises
- What is the impact for Mr. Gauthier of having received contradictory information about the start of his treatments after surgery?
- How does the way in which Mr. Gauthier participates in care decisions make his care experience more positive?
- How does the way in which Mr. Gauthier’s family members are involved make his care experience more positive?

**C) End of the focus group discussion**

- Thanks to everyone for their participation
- Reminder about confidentiality
- Offer to send participants a summary of the discussion if they want it

**Focus group summary**

**A) Objective:** Based on a summary of the data collected in the focus groups with professionals and with patients, a validation discussion will be conducted with persons designated by the local groups. These persons should be representatives of the physicians, nurses, other professionals, managers, and patients.

**B) Procedure (60 minutes)**

The discussion on the summary could be conducted as a video conference or teleconference with the research team, the facilitator, and the designated local representatives. The aim of this discussion will be to validate whether the summary accurately reflects the tone of the discussions, to confirm the CMOs identified in them, and to agree on the lessons that can be learned from them.
